# Faculty professional development and career advancement through Student Evaluations of Teaching (SETs)

**DOI:** 10.1186/s12919-026-00392-2

**Published:** 2026-09-14

**Authors:** Maria Zulema Cabail, Roslyn N. Crowder, Maitreyi Das, Verónica A. Segarra

**Affiliations:** 1 Biological Sciences Department, State University of New York (SUNY) Old Westbury, Old Westbury, Long Island, NY 11568 USA; 2 Department of Biology, Stetson University, DeLand, FL 32723 USA; 3 Biology Department, Boston College, Chestnut Hill, MA 02467 USA; 4 Departments of Biological Sciences and Chemistry, Goucher College, Baltimore, MD 21204 USA

**Keywords:** Student evaluations of teaching, Student evaluations, Faculty professional development, Three-voice framework, Peer-evaluation of teaching, Teaching portfolio

## Abstract

New faculty members starting to develop their pedagogy often realize quickly that student evaluations of teaching (SETs) are essential components of a faculty member’s progression through the academic ranks. The degree to which these evaluations influence one’s advancement up the academic ladder varies depending on the type of institution. At the same time, these artifacts can also help inform an educator’s pedagogical practice and refinement of a course over time. This reflective commentary article draws on relevant scholarly literature and our lived experiences as scientist educators to examine the uses and limitations of SETs. Although SETs are widely used to evaluate teaching, there is limited guidance on how faculty can systematically use these data to inform professional development and career advancement. To address this gap, we propose a six-step SET analysis and reflection strategy comprising: (1) initial SET review, (2) close SET review, (3) data extraction and analysis, (4) reflection, (5) identification of changes to teaching practice, and (6) development of an action plan for implementing those changes. This structure will help better prepare faculty members to utilize student teaching evaluations for professional development and advancement.

**Supplementary Information:**

The online version contains supplementary material available at https://doi.org/10.1186/s12919-026-00392-2.

## Background

SETs are a longstanding institutional tool for assessing faculty. There is an accumulating body of literature documenting the flaws of only using SETs to evaluate a faculty member's teaching effectiveness. In this commentary article, we briefly review this accumulating body of literature and discuss a structural approach to effectively using SETs as a tool, among other supplemental resources, to foster faculty professional development. In this context, SETs can be a powerful resource in faculty professional development if used with the proper scope.

### A historical context for student evaluations of teaching

Formal SETs in the United States originated in the 1920s as a way to provide instructors with feedback and help them improve their teaching [1]. Educational researchers at Purdue University and the University of Washington developed early student evaluation instruments with the intention that the feedback provided through these instruments be kept confidential and used only by instructors [2]. However, by the 1950 s, SETs were being widely utilized by university administrators to assess faculty members, often at the behest of student groups.

The widespread use of SETs to make personnel decisions did not begin until the 1980s. By the 1990 s, 86% of colleges in the U.S. were using SET data to inform decisions about faculty hiring, salaries, and promotions. By 2010, that number had risen to 94% [3]. This shift was accompanied by an increased emphasis on standardized measures of student learning and a growing demand for accountability from external stakeholders such as accreditation agencies, institutional boards of trustees, and employers [4].

The use of SETs for high-stakes decision-making has always been controversial. Faculty have historically preferred that evaluation instruments be locally developed and used primarily for professional development purposes. Concerns about the validity, reliability, and fairness of SETs, especially when used for comparative purposes, were being voiced as early as the 1970s. As a result, faculty support for SETs waned as their use in personnel decisions became more common [1, 2].

Despite concerns about the impact of SETs on academic freedom and the quality of teaching, their use remains widespread today [5]. This is due in part to the fact that no other method of evaluating teaching has gained widespread acceptance [6]. However, as the body of research documenting bias and other issues with student evaluations grows, universities are increasingly being pressured to reconsider their use [5].

### Bias in student evaluations of teaching

Many factors unrelated to teaching effectiveness can influence SETs. Instructor identity—such as gender, race, ethnicity, accent, sexual orientation, or disability status—can affect results due to equity bias [5, 6]. Research indicates that male instructors are more likely to be perceived as competent, brilliant, or organized, whereas non-white instructors tend to receive lower SET ratings than their white colleagues [2, 5]. Moreover, research suggests that women and faculty members of color receive more negative comments related to personality, appearance, mannerisms, competence, and professionalism [5].

Class size also contributes to measurement bias, with instructors of larger classes often experiencing a "large class penalty" [5, 6]. Course characteristics—including class time, whether the course is required or elective, course difficulty, and discipline—further impact SET results [5]. Disciplines vary in how easily they engage students; for example, generating interest in social psychology may be less challenging than in statistics [2]. Similarly, teaching quantitative courses often presents additional challenges [7].

A recent study found that Science, Technology, Engineering, and Mathematics (STEM) students tend to have lower grades and Grade Point Averages (GPAs), primarily due to more stringent grading in STEM courses [8]. This grading disparity may partially explain why SET ratings in STEM courses are generally lower than in non-STEM courses. Given these biases, administrators should avoid relying on SETs as the sole measure of teaching effectiveness. SETs provide student feedback and should be understood as reflections of student perception and experience, not as direct measures of teaching effectiveness or student learning [5, 9, 10].

## Things to consider before students complete SETs

Preparation for SETs must begin before a course is taught. The preparation needed for successful SET administration can be built into the instructor’s process of preparing course materials, such as the syllabus, and spaces, including the course site and learning management system of choice. The instructor needs to know what students will be asked in SETs. For this reason, an essential initial step to prepare is to obtain a list of the questions or prompts that will be asked of students as part of the SET. An institutional representative, such as a department chair, director of the center for teaching, or dean of faculty, should be able to assist in obtaining this information. Once this is obtained, the instructor can use backward design to ensure that each of the elements the students will be assessing is aligned with the course policies and practices. In some cases, institutions may give the course instructor the option of customizing questions on their course SETs. Table 1 outlines sample questions that students may be asked in SETs and ideas instructors can consider to maximize alignment between the course structure and student assessment. While the institution is responsible for deploying and administering the end-of-semester SET, instructors can decide to craft an anonymous assessment mid-term so that the course can be adjusted before the conclusion of the course nears. Mid-term assessments can be set up using a survey management system like Qualtrics or Google Forms and can be as short as desired. One effective strategy for a mid-term check-in is to ask students two reflective questions: what aspects of the course are going well, and what aspects they would like to see changed. Ideas for additional mid-term survey items can be found in Additional File 1 (Sample Midterm Student Survey). The home institution might also have mid-term assessment ideas that might be of interest to the instructor.

**Table 1:** Sample questions that students may be asked in SETs and ideas instructors can consider to maximize alignment between course structure and student assessment of the course

Student evaluation instrument question or prompt	Reflection questions or prompts for the instructor to consider before teaching a course
How would you rate the clarity of grading criteria for tests and assignments?	Does the course syllabus contain clear grading criteria for tests and assignments, including rubrics? Can students easily access these resources, for example in the course learning management system (LMS)?
How would you rate the degree to which this course's laboratory sessions helped you learn?	Does the course syllabus and lab materials help students understand how the laboratory activities help them attain the course objectives? Will students be reminded of this connection throughout the academic term?
How would you rate the extent to which you received feedback on assignments?	Do students understand how the instructor plans to provide them with feedback on assignments?
How would you rate the instructor's enthusiasm in class?	How will the instructor express to students their enthusiasm for the subject and for the learning community they are co-creating?
How would you rate the respect the instructor showed you?	How will the instructor show students respect? To what extent can classroom management and interpersonal skills help the instructor show their students respect?
How would you rate the degree to which the instructor followed the syllabus?	Does the course syllabus note that it is subject to changes? When changes are needed, how and when will students be notified? Are there syllabus elements that will likely not change, for example, assessment dates? It might be beneficial for students to know which syllabus elements are not likely to change.
How would you rate the degree to which the instructor was prepared for class?	What systems can the instructor put into place to ensure that they will be in a position to arrive to class on time with all the materials that they need?
How would you rate the timeliness with which the instructor returned your graded materials?	Do students know how soon to expect graded work back?
How would you rate the instructor's concern for your progress in this course?	How will students know that the instructor cares about their learning and progress in the course?

Generally, at the tail end of a course, students will be given a period to complete SETs, which will most often be administered online. Higher response rates in SETs can yield more complete information on the impact of a course and the instructor's efforts. While the literature suggests measures to foster student participation in SETs, including increasing transparency about how SET feedback is used, when empirically tested, the effect of these measures is not always clear [11, 12]. Table 2 lists ways that can be used to foster student participation in SETs [13–21]. It is important to keep in mind that, just like individual instructors have pedagogies that resonate with their values and are available to them, not all of the strategies listed in Table 2 will be synergistic with an instructor’s teaching philosophy and ways of facilitating their classroom dynamics.

**Table 2:** Strategies to foster student participation in end-of-semester SETs. Strategies have been divided into themes or areas—Class time, Communication, and Grading Tools. It is often the case that institutional Centers for Teaching and Learning will have additional recommendations and considerations. While an instructor can strive to foster student participation in SETs, it is paramount to remember that it is likely the case that students are not required to complete them–students have a choice. Information was synthesized from the literature [13–21]

Strategy	Considerations
**Class (Time) tools**	
Set aside time during the class period for students to complete SET	Is it appropriate for the instructor to remain in the room? Does the SET need to be proctored? Institutions likely have guidelines that instructors are expected to follow when administering SETs. A colleague or teaching assistant can be recruited to proctor SETs.
**Communication tools**	
In-class reminder	When do you encourage students to complete the SET? The institution will likely have a window of time during which the SET is available for students to take. This can help the instructor plan when to provide an initial reminder and whether to provide additional ones. The instructor may want to share with students how student evaluation feedback is used to modify the course and teaching practice. Examples of past changes could be offered to students so that they understand the value of their feedback. Individual/personal reminders may be more effective than sending a message to the whole class.
Email reminder	
Announcement on Course Learning Management System	
Include SET information in the course syllabus	Instructors may consider including SETs as an activity in the course calendar. In this case, care should be taken to ensure students understand whether they have a choice to complete them or not.
**Grading tools**	
Possible extra credit or bonus points, as determined by the instructor, if a certain percentage of students complete SETs	How high the threshold is set (for example, 100% versus 75%) might influence whether students see this as an incentive.
**Engagement tools**	
Built-in formative course assessments before end-of-semester SETs	Some studies indicate that instructors who conducted mid-term evaluations (see Appendix 1 for sample, home institution might also have mid-term assessment recommendations) and implemented associated changes experienced an increase in end-of-semester SET completion.
**Institutional tools**	
Early access to final grades	Institutions may offer students incentives for completing their SETs, including early access to final grades and/or the registration process.

While SETs can provide the student perspective on a course, they do not provide a complete picture. For this reason, instructors should supplement SET data with peer evaluation and reflections on their teaching. Additionally, instructors must develop a strategy or workflow for analyzing and reflecting on their SET data once it is available. Despite their flaws, SETs can still play a role in a comprehensive evaluation system [5, 9].

## SET analysis and reflection strategy for professional growth

SETs have become a ubiquitous element of higher education, serving as a common mechanism for gathering feedback on instructional effectiveness [10]. While debates regarding their validity and potential biases persist [2, 5, 9], a thoughtful and systematic analysis of student feedback, particularly qualitative comments, can provide a valuable iterative strategy for educators seeking continuous professional growth. By actively engaging with student voices and refining their instructional practices over time, instructors can strive to become more effective and responsive teachers.

Incorporating student feedback into an iterative cycle of improvement requires a fundamental understanding of the nature of SETs. These evaluations, which have a long history in academia [1], typically consist of both quantitative ratings and qualitative written responses. While numerical scores can offer a broad overview of student perceptions, written comments often yield richer, more nuanced insights into the student experience, pinpointing specific areas for pedagogical refinement [10]. However, it is crucial to approach these comments with a critical lens, acknowledging the well-documented potential for biases related to instructor gender, race, and other characteristics [5, 9].

**Fig. 1:**
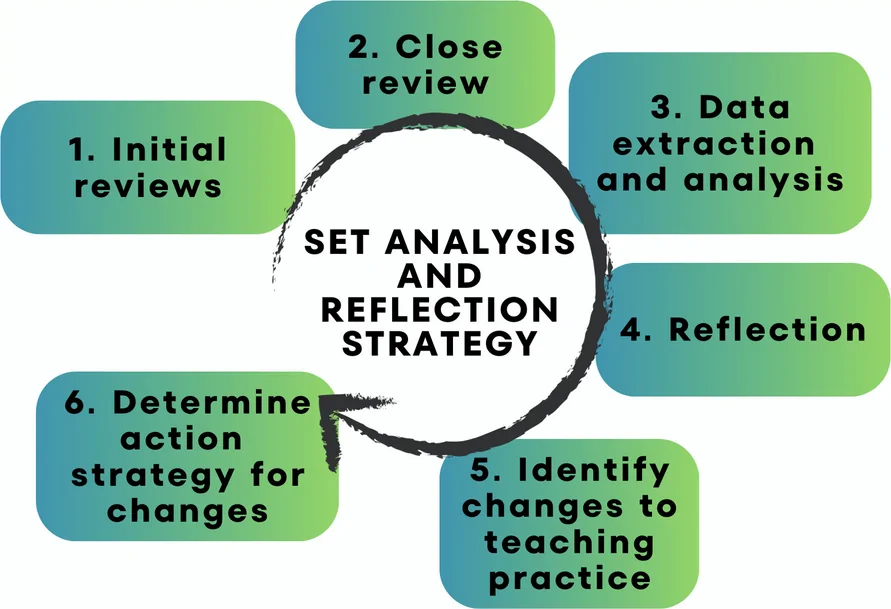
SET analysis and reflection strategy. This diagram illustrates one of the iterative approaches faculty can take when reviewing and analyzing their SETs. The process involves a desired number of initial reads or reviews in which the faculty reads SETs for content only. These initial reviews can then be followed by a close review with the ultimate goal of extracting evaluation data for analysis and reflection to determine desired changes to a course and/or the instructor’s teaching practice. Part of the process will be to determine the actions necessary to implement the desired changes

To thoroughly review and analyze SETs, faculty members must read, process, and reflect on the scores and comments that students have shared. To effectively utilize student feedback for professional development, instructors can adopt a systematic and iterative analysis strategy. This process begins with the regular collection and careful review of evaluations at the conclusion of each academic term. We outline a SET analysis and reflection strategy in Fig. 1, with detailed commentary about each step below.

### Step 1–Initial SET review

It may be tempting to ignore reading SETs, but to utilize SETs for professional development and growth, SETs should be read more than once. The process ideally involves several fast initial readings or reviews, during which the faculty reads SETs for content and first impressions only. It may be helpful to allow for time, for example, days or weeks, between initial readings. Spacing out readings like this may help faculty manage the discomfort that may arise from SETs [22].

### Step 2–Close SET review

Close readings might take longer to complete than initial readings. Close review might also involve note-taking and reflection, allowing for the identification of trends that are captured in SET data. This detailed look at SETs will set the stage for data extraction and analysis that will follow.

### Step 3–Data extraction and analysis

Once faculty complete their SET reviews, they can extract data from them for meaningful deliberation and critical evaluation of their teaching. Once SETs have been made available to faculty, often some time after final grades have been submitted, they can download a detailed report of the results, likely as a PDF file. Depending on the system used by the institution, faculty may also have the option to download the raw data as a spreadsheet, making it easier to extract the data from the report for analysis. However, it will often be the case that the faculty will have to manually extract numerical data and/or student comments from the PDF file provided into a desired file, most likely a spreadsheet, for analysis and longitudinal tracking. Having this data on a spreadsheet, including benchmarks for schools, colleges, or departments at the institution, will allow the faculty member to plot, graph and construct tables that show their trends over time in comparison to reference values. Manually taking data from a PDF to a spreadsheet can be a cumbersome task, so it is recommended that the faculty develop a routine to do this as they get ready to close out the term and start another. This is especially the case if the faculty member is pre-tenure because their analysis and associated graphs and tables can then be included in their annual review documents and tenure and promotion dossiers. Table 3 highlights key points to be considered when extracting data and noting SET trends through analysis. Some might even consider using generative artificial intelligence (AI) to help analyze their SET data [23]. While AI technology has great potential, it is still developing, so one should exercise caution when using it for SET analysis.

**Table 3:** Tips for analyzing SET data. Once data has been extracted from a SET report and placed into a spreadsheet, faculty can analyze their data in a variety of ways. Institutions might have suggestions on specific analysis to perform. In this table, we share suggestions for faculty to try with their data, along with associated considerations (right column). The suggestions are classified into data curation and usage recommendations

Analysis tip	Notes and considerations to keep in mind
**Data curation**	
Prepare SET numerical score spreadsheet	Numerical values from SET reports can be transferred to a spreadsheet for analysis and longitudinal tracking. To minimize the number of spreadsheets that are used, the data can be continuously recorded on one file with different tabs for courses or terms of interest.
Data should include SET response rates	The higher the SET response rate for a course, the more representative the data will be of the course. Keeping track of response rates will help the instructors ascertain which SET reports are more likely to reflect the most complete assessment of their teaching. Response rates can be easily calculated as percentages.
Data should include benchmark values	SET reports will include institutional benchmarks–overall values for the institution, school/college/division, or department. It is useful to note how close your values are to those benchmarks and the potential reasons why.
Update SET data spreadsheet at the end of each term	Manually taking data from a PDF report and copying it to a spreadsheet can be a tedious and time-consuming task! Faculty should consider doing this piecemeal, routinely doing this as they get ready to close out the term and start another.
Statistics	Having data on a spreadsheet will facilitate calculations such as averages and standard deviations. Statistical analysis of the data will in turn allow the faculty to identify outliers, if present.
Consider disaggregating data by categories	Questions in a SET are often categorized into sections. Example categories include the instructor's preparedness, the academic support provided by the instructor, and the feedback provided by the instructor. Instructor strengths and weaknesses will likely become evident when analyzing the results by categories instead of overall values.
Identify student comments that illustrate the numbers, keeping in mind that comments and numbers may not always align	SET reports will also include student comments. As instructors perform their analysis, it is useful to select student comments that bring the numerical trends in the data to life. These can be cited as quotes in documents (see below) to enhance their impact. It is essential to recognize that student comments can also reveal their unconscious biases.
**Uses**	
This data can be cited in faculty annual review, tenure & promotion documents, and teaching portfolios	Figures or tables can be constructed to serve as evidence and back up points of discussion.
Use data to advocate for resources or changes to courses	Student voices as reflected in SETs can be used as evidence for the need of a resource or change that is not under the control of the instructor.

Faculty should also carefully analyze the student comments in the SETs. Rather than focusing on isolated remarks, the emphasis should be on identifying recurring themes and patterns that emerge across multiple evaluations and courses over time (Tables 3 and 4). For example, consistent feedback about unclear explanations or an overwhelming workload may highlight areas in need of attention [2]. It is essential to prioritize feedback that is representative of the majority of students, recognizing that outlier opinions, while sometimes strongly voiced, may not reflect the overall class experience [10]. See Table 4*,* under *Factors that are likely outside the control of the instructor,* for additional context. Furthermore, actionable feedback, which offers specific suggestions for improvement, should be given particular consideration. A comment such as “incorporate more real-world examples” provides a clearer pathway for instructional refinement than a vague critique of an instructor’s style.

**Table 4:** Sample student evaluation comments and associated instructor commentary on ideas conveyed in student comments. Students' comments have been divided into themes, shown in bolded font. Student comments that are from the same class, seemed contradictory, and were shared by different students are shown in *italics* to highlight how students in a course can have a range of preferences and thoughts

Student comment	Considerations
**Assessments and class time tools**	
I wish there were more quizzes before each test so that the information could be broken down into smaller segments.	Can homework, quizzes, or problem sets be built into the class between exams to help students assess their knowledge of the content? These can be posted on the LMS of interest to facilitate grading.
The strengths of the course include the review sessions and review documents for the quizzes and tests.	Can the instructor leverage the course elements that students cite as strengths? Be sure to keep these elements in the course!
Some of the tests were difficult because of the wording of the questions and similar options in the multiple choice questions.	Can the instructor seek feedback from colleagues to ensure that the test questions are assessing students’ understanding of the content of interest? Were there test questions that most students failed, potentially indicating that the question may not have been aligned with the learning objectives?
*The use of PowerPoint slideshows and the ability to download them were noticeable strengths.*	It is unlikely that all students will like all pedagogical approaches an instructor uses in a course. It is not rare for student comments on an evaluation to seemingly contradict one another. This highlights the importance of using multiple pedagogical approaches to teaching content in a course.
*I had a hard time understanding the material based on the PowerPoints.*	
The teacher’s enthusiasm and love for the subject rubbed off on me and made me excited to learn more.	Not all instructors show enthusiasm in the same way, but hopefully instructors can find ways to authentically exteriorize it to students.
The teacher’s enthusiasm towards certain topics made listening to the lectures more interesting.	
It is much easier to learn from you in smaller more one-on-one situations as opposed to the large lecture room.	While instructors find different ways to make themselves available to students, including, but not limited to, office hours and exam review sessions; one-on-one interactions allow students to get to know the instructor better and learn from them in different ways.
Sometimes when you talk to students your tone of voice is condescending.	Individuals have their own ways of communicating and talking. One thing that will help students get to know you is to create opportunities for them to interact with you outside of the classroom. Consider low-stakes assignments that require students to interact with you one-on-one for feedback or course-related tasks. Also, consider arriving early to class to interact with students informally as they arrive in the classroom. When these interactions are built into a course experience, it is less likely that an instructor’s tone will be misunderstood.
**Factors that are likely outside of the control of the instructor**	
I think having the same professor for both lecture and lab would have helped tremendously.	Can the instructor ask the department to teach the same populations of students in both lab and lectures? Sometimes this is not possible. In these cases, it can be helpful to share with students the efforts in place to keep course components aligned.
My only complaint is that it was tough to stay focused in class with it being at 8 am.	Can the instructor consider utilizing more engaged pedagogies for morning courses, to help students stay focused and on task?
The room was uncomfortable and made it somewhat difficult to focus.	Can the instructor ask for a different classroom assignment?
The teacher had an accent.	Instructors can consider, as part of the beginning of classes routines, sharing with students information about their story and positionality. This will help the students establish a connection with them and see them as an individual.
**Factors that highlight students’ growth mindset**	
It was challenging in the beginning but then I learned how to study better for exams and I did everything I could to review the content, I also needed to obtain an outside tutor.	Comments that highlight students persevering and adapting to taking a challenging college course can serve as positive reinforcement to the instructor. They can also provide the instructor with insight as to what strategies are used and developed by students for success in the course.
I have learned good techniques for studying that actually work.	

#### Cautious interpretation

SET results should be interpreted with caution. While student feedback provides direct insights into the learner experience, it is important to contextualize this information by considering factors such as course level, subject matter, and student demographics [5]. They are not designed for direct comparison across faculty or disciplines, but rather to observe a faculty member's own teaching "trajectory" over time, ideally within a single course. In addition, administrators should look at the overall distribution of ratings, or the median/modal response, rather than solely the mean, as a few low ratings can skew the mean. [5, 10]. SETs should not serve as the sole metric for professional growth. Integrating feedback from peer observations, mentorship, and self-reflection offers a more holistic perspective on teaching effectiveness [9, 10].

### Step 4 –Reflection

As instructors accumulate years of evaluation data, a longitudinal perspective becomes invaluable. Analyzing trends over time can reveal areas where prior adjustments have been successful and highlight new or persistent challenges [24]. This historical lens allows for a more informed approach to pedagogical experimentation. Based on identified themes, instructors can implement targeted changes in teaching methods, course design, or assessment strategies [9]. Subsequent evaluations can then assess the impact of these modifications on student learning and engagement [10]. Additionally, recognizing and consistently reinforcing positive feedback can help instructors build upon successful teaching practices and explore avenues for further enhancement [24]. One effective way for instructors to develop awareness of their teaching practices is by maintaining a reflective teaching journal [25–28]. When keeping a teaching journal, instructors can compare their recorded reflections and observations to SET data trends. Through ongoing reflection, faculty can set meaningful goals, track their progress, and adapt their strategies to enhance student learning outcomes [2, 9, 29]. As an aid, we offer Additional File 2: Table 2* Faculty SET reflection table*.

### Step 5 –Identify changes to teaching practice

Once the faculty has analyzed their SET results and reflected on them, they can identify changes that they would like to make to a course and/or their teaching style or practice. These changes should align with the data. To illustrate alignment, Table 4: *Sample student evaluation comments and associated instructor commentary on ideas conveyed in student comments* lists sample student comment themes and changes that they might inspire in a course. Identified changes should also leverage an instructor’s strengths and bolster or address the weaknesses they wish to improve. If the instructor wishes to make more than a few curricular changes, the desired changes must be prioritized, as it is not recommended to implement too many changes at once [30, 31]. Over the course of an academic career, the focus of instructional improvement may evolve. Early on, addressing foundational aspects such as clarity and organization may take precedence [24]. With more experience, instructors can use student feedback to refine more nuanced elements of teaching, such as fostering critical thinking or promoting inclusivity [24]. Even long-serving faculty members can benefit from this iterative process by addressing persistent challenges or experimenting with significant pedagogical shifts. Depending on the institution, some of these processes may be carried out in collaboration with offices like institutional teaching and learning centers [10]. This highlights the idea that faculty should keep in mind that they are not alone in improving their teaching practice. In addition to institutional teaching and learning centers, institutional resources that may be available to faculty include coaching by departmental members, for example the chair, and/or a faculty advocate.

### Step 6 –Determine action strategy for changes

Some of the desired changes might be addressed with simple actions such as modifications to the syllabus or course structure. Some others might require substantial labor to implement, for example, revamp a course’s lab component, implement engaged student pedagogy, or design new course resources. It would be difficult to effectively assess the impact of each change if the instructor attempts to implement several changes simultaneously. Therefore, it is advisable that instructors take stock of how much labor a set of changes will take to implement, and only try to address as many changes as is reasonable to implement during one term [30, 31].

It is important to approach student evaluation comments with certain caveats in mind. Over-reliance on SETs as definitive measures of teaching effectiveness should be avoided [32]. Instructors should also be cautious about overinterpreting minor fluctuations in scores [10]. Recognizing that some criticisms may be idiosyncratic or stem from factors beyond the instructor's control is equally important [9]; see Table 4*,* under *Factors that are likely outside the control of the instructor,* for additional considerations. Finally, the goal of analyzing student feedback should be professional growth rather than direct comparison with colleagues.

When analyzed systematically and iteratively, student evaluation comments offer a powerful tool for continuous professional development in teaching. By actively listening to and thoughtfully responding to student voices, while remaining mindful of the inherent limitations and potential biases of SETs, educators can embark on a journey of lifelong learning, striving to become more effective and impactful teachers throughout their academic careers. Support from institutional, departmental, and peers can facilitate an educator’s holistic growth and development.

## Leveraging SETs holistically

Although this commentary article focuses on SETs, teaching effectiveness is typically evaluated using multiple sources of evidence. To advance towards a more holistic evaluation of teaching (HET) and effectively leverage SETs, it is essential to integrate multiple measures of instructional effectiveness, acknowledging the limitations and benefits of each. A comprehensive HET approach collects evidence from multiple sources to construct a well-rounded picture of an instructor’s performance and development over time. This multi-modal assessment provides a more accurate evaluation than any single measure, as inconsistencies or limitations in one source can be balanced by strengths in others, particularly when the imperfections differ across measures [5, 9, 32]. This strategy is commonly referred to as the "three-voice framework" and typically incorporates feedback from students, peer reviewers, and the instructor’s own self-reflection [29]. Together, these perspectives offer a contextualized, more balanced, and equitable foundation for evaluating teaching quality and supporting continuous improvement. Below we present two subsections to illustrate how SETs can be interpreted in conjunction with complementary forms of teaching evidence.

### SETs as part of comprehensive teaching portfolios

Teaching portfolios offer faculty an opportunity to present a comprehensive record of their instructional practices. In addition to peer-review commentaries and SET analyses, these may include detailed descriptions of course design, pedagogical goals, key themes, reading lists, assessment tools (such as exam questions), and instructional materials like PowerPoint presentations and assignments [2, 5, 9, 10]. Teaching portfolios can also include data on Student Learning Outcomes, including tools such as pre- and post-assessments. These tools offer evidence of student learning. While valuable for evaluating instructional effectiveness, these outcomes often show little to no positive correlation with SETs [29].

### Peer evaluation of teaching as a complement to SETs

While the specific term used for this may vary by the institution, in general, peer evaluation of teaching involves faculty members observing a colleague’s class and providing constructive feedback on instructional methods, classroom engagement, course materials, and assessment methods, with the goal of supporting and improving teaching effectiveness. To be effective, these observations and evaluations should be structured, consistent, and conducted over time [29]. Teaching peers bring valuable disciplinary knowledge and pedagogical expertise, allowing them to provide constructive feedback that students may not be equipped to offer. It is important to *plan ahead* for these peer observations and assessments of teaching. Planning ahead allows for constructive feedback that is structured and supported by formal observation forms and pre-/post-observation meetings [33]. These forms and associated meeting guidelines are often provided by the institution, especially since the process might be part of a faculty member’s promotion and tenure or annual evaluation. A structured process, in turn, ensures that feedback focuses on meaningful aspects of teaching rather than superficial elements and helps ensure that feedback is both constructive and actionable [29]. Peer evaluations of teaching also allow for increased reliability and contextualization of teaching assessments. Peer evaluations enhance the reliability of overall teaching assessments by incorporating multiple sources of evidence and disciplinary expertise [29]. While peer evaluations can carry their own biases, they are less susceptible to systemic biases associated with SETs, such as those related to an instructor’s gender, race, or appearance. Combining student and peer feedback can help balance out these limitations and mitigate the bias in SETs. Peer reviewers are also better positioned to assess elements that SETs typically overlook, such as course rigor and workload expectations [10]. A well-designed peer evaluation process can help build faculty trust in the evaluation system by addressing concerns that may arise from SETs–including low response rates, limited constructive feedback, and bias [29]. When peer evaluations are framed as developmental tools rather than administrative checkboxes, they promote continuous improvement in teaching.

## A role for institutional support

Effective use of SETs requires not only thoughtful reflection by instructors but also institutional practices that support fair and meaningful evaluation. Therefore, we briefly discuss some recommendations for academic leaders. Evaluations that track growth over time foster a culture of reflective practice and professional development, rather than one of compliance or punitive judgment [1, 10, 29]. By implementing structured, intentional, and ongoing peer evaluation practices, institutions can cultivate a more robust and credible framework for evaluating teaching, one that complements and contextualizes student evaluations to offer a complete picture of instructional effectiveness. Institutions should actively encourage student participation by providing meaningful incentives, such as early grade release for those who complete evaluations, which can significantly improve response rates and data quality. When making decisions about promotion, tenure, and retention, institutions should prioritize numerical evaluation scores over qualitative responses to specific questions, as quantitative data provides more standardized and comparable metrics across different instructors and courses. However, these numerical scores must be contextualized to account for inherent biases related to class size, course complexity, and student demographics—for instance, introductory courses often receive different evaluation patterns compared to specialized upper-level seminars, and courses with heavier workloads may face rating penalties unrelated to teaching quality. Rather than relying on single-year snapshots, institutions should analyze teaching evaluations longitudinally, examining trends and improvements over time to gain a more comprehensive understanding of an instructor's development and consistent performance. Additionally, institutions should consider implementing peer evaluations from colleagues, self-reflection components that allow instructors to provide context for their teaching approaches, and student learning outcome assessments that measure actual educational effectiveness rather than satisfaction alone. These multifaceted approaches, combined with proper statistical adjustments for course-specific variables and clear communication about evaluation purposes, can transform teaching evaluations from potentially biased popularity contests into meaningful tools for professional development and institutional decision-making [5, 9, 32].

## Concluding remarks

With every course they teach, instructors have the opportunity and responsibility to refine their teaching methods and customize content to meet the needs of their students, collectively helping train the next generation of scientists [34]. An essential aspect of this refinement is for instructors to use the tools at their disposal, including SET data, to ensure their own growth and development as educators. However, in the vein of the old adage, "you manage what you measure," or "you are what you measure," SETs can only provide as much information as they actually capture. SETs, as constructed by the institution and its administrators, may not overtly put forth whether a course’s learning objectives are being effectively met. By relying on SETs as the main cornerstone of faculty teaching assessment, we lose the effective angles that other methods can provide. For this reason, SETs must be used intentionally and reflectively, always in combination with tools like peer-evaluation of teaching. In this way, SETs can become an invaluable tool that helps instructors as they strive to teach and serve students effectively.

## Supplementary Information

Additional file 1: Sample Mid-term Student Survey: this file contains a sample mid-term student survey that instructors can use as a model and adapt as needed.

Additional file 2: Table A2. Faculty SET reflection table: this table is a sample template for faculty to reflect on their SETs for a particular course and distill the main themes and desired changes for the next iteration of a course of interest.

## Data Availability

Not applicable.

## Abbreviations

GPA: Grade Point Average
LMS: Learning Management System
STEM: Science, Technology, Engineering, and Mathematics
SET: Student Evaluation of Teaching
