# Supplementary material for: Faculty professional development and career advancement through Student Evaluations of Teaching (SETs)
Source: BMC Proc. 2026 Sep 14;18(Suppl 1):34. doi: 10.1186/s12919-026-00392-2 (PMC13573320; doi:10.1186/s12919-026-00392-2)
Supplement: Supplementary file 1 [file 12919_2026_392_MOESM1_ESM.docx]

**Appendix 1**

**Sample Mid-term Student Survey**

The pace of new topics in this course so far is (select one):

o	Too fast

o	Too slow

o	Just right

The number of assignments I have been asked to complete in this course so far is (select one):

o	Too much

o	Too little

o	Just right

The exam(s) in this course so far have been (select one):

o	Easier than I thought

o	Harder than I thought

o	About what I expected

o	Other [text box]

In this course, I would like more of the following (select all that apply):

o	Group work

o	In-class practice exercises

o	Homework

o	Other [text box]

To prepare for the next exam and earn the grade I want, I need to do the following (select all that apply):

o	Increase my study time

o	Start studying earlier

o	Attend tutoring sessions

o	Join a study group

o	Change nothing

o	Other [text box]

Which resource has been the most helpful for you while studying for this course (select all that apply)?

o	YouTube videos

o	PowerPoint slide decks

o	Learning objectives

o	Note taking

o	Other [text box]

Do you have any suggestions that could help you get more out of this course? [text box]
