# Supplementary material for: Faculty professional development and career advancement through Student Evaluations of Teaching (SETs)
Source: BMC Proc. 2026 Sep 14;18(Suppl 1):34. doi: 10.1186/s12919-026-00392-2 (PMC13573320; doi:10.1186/s12919-026-00392-2)
Supplement: Supplementary file 2 [file 12919_2026_392_MOESM2_ESM.docx]

**Appendix 2: Table A2**. *Faculty SET reflection table*. This table is a sample template for faculty to reflect on their SETs for a particular course and distill the main themes and desired changes for the next iteration of a course of interest. This template can easily be adapted to include additional areas (as rows and/or columns) for an instructor to reflect on or note.

|  | COURSE: | TERM: |
| --- | --- | --- |
|  | **COURSE COMPONENT** | |
|  | **LECTURE** | **LAB** |
| What worked? |  |  |
| What did not work? |  |  |
| Changes to be made for the next iteration of the course |  |  |
| Additional comments or concerns |  |  |
